# Persuasive Features of Scientific Explanations: Explanatory Schemata of Physical and Psychosocial Phenomena

**DOI:** 10.3389/fpsyg.2021.644809

**Published:** 2021-09-06

**Authors:** Jordan Richard Schoenherr, Robert Thomson

**Affiliations:** ^1^Department of Psychology, Concordia University, Montreal, QC, Canada; ^2^Army Cyber Institute, United States Military Academy West Point, West Point, NY, United States; ^3^Behavioral Sciences and Leadership Department, United States Military Academy, Highlands, NY, United States

**Keywords:** explanation, reasoning, metacognition, overconfidence, scientific explanations, scientific communication, folk theories

## Abstract

Explanations are central to understanding the causal relationships between entities within the environment. Instead of examining basic heuristics and schemata that inform the acceptance or rejection of scientific explanations, recent studies have predominantly examined complex explanatory models. In the present study, we examined which essential features of explanatory schemata can account for phenomena that are attributed to domain-specific knowledge. In two experiments, participants judged the validity of logical syllogisms and reported confidence in their response. In addition to validity of the explanations, we manipulated whether scientists or people explained an animate or inanimate phenomenon using mechanistic (e.g., force, cause) or intentional explanatory terms (e.g., believes, wants). Results indicate that intentional explanations were generally considered to be less valid than mechanistic explanations and that ‘scientists’ were relatively more reliable sources of information of inanimate phenomena whereas ‘people’ were relatively more reliable sources of information of animate phenomena. Moreover, after controlling for participants’ performance, we found that they expressed greater overconfidence for valid intentional and invalid mechanistic explanations suggesting that the effect of belief-bias is greater in these conditions.

## Introduction

Our ability to comprehend the quality of scientific evidence is critical to navigating the modern world: whether in terms of assessing the prescriptions of clinicians, determining the likelihood and extent of global warming, the function and output of algorithms and artificial intelligence, or understanding the culpability of an accused criminal. For instance, despite the recent outbreak of COVID-19, polling indicated that 66% of Americans were not concerned that the virus would directly affect them (Schulte, 2020). Moreover, disinformation and misinformation led to further confusion over the nature of the virus and influenced national responses during the pandemic (Emmott, 2020; Robins-Early, 2020; Schulte, 2020). Given significant discrepancies between beliefs held by scientists and the general public on issues including climate change, vaccines, the theory of evolution, and genetic modified organisms (e.g., Pew Research Center, 2015), and certainty with which scientific and health beliefs are held (Drummond and Fischhoff, 2017; Motta et al., 2018; Park et al., 2020), there is a need to ensure that expert knowledge is intelligible to those outside of their respective research communities (e.g., Kolstø, 2001; Zimmerman et al., 2001; Zhu et al., 2011; Scharrer et al., 2012; Thomm and Bromme, 2012; Kraft et al., 2015).

In the present study, we examine features of causal explanations (e.g., Lombrozo and Vasilyeva, 2017) that might interfere with the acceptance of logically consistent arguments used in scientific explanations. Using methods adopted from the reasoning (e.g., Evans et al., 1983; Sa et al., 1999; Newman et al., 2017) and persuasive communication literatures (e.g., Cialdini, 1984/2007; Chaiken and Trope, 1999; Bohner and Dickel, 2011), we developed a “minimal explanation” paradigm that manipulated features of explanatory schemata from two different domains (folkpsychology/folkbiology and folkphysics; e.g., Spelke and Kinzler, 2007; Gelman and Legare, 2011). By manipulating the animacy of the explanandum (animate or inanimate natural phenomena), the explanans (intentional or mechanistic explanations), as well as the source of the information (e.g., ‘people’ or ‘scientists’), our results revealed that participants’ maintained heuristics based on their prior beliefs which biased their responses. By statistically controlling for participants’ performance when assessing response confidence (e.g., Ziori and Dienes, 2008; Schoenherr and Lacroix, 2020), we examined subjective perception of certainty for intentional and mechanistic explanations, with evidence suggesting that participants experienced more overconfidence for valid intentional explanations and invalid scientific explanations.

### Features of Explanations

Explanations have been a persistent focus of the philosophy of science (e.g., Hempel and Oppenheim, 1948; Craik, 1952; Salmon, 1989; Woodward, 2003; Strevens, 2008; Glennan, 2009; Craver, 2014). However, philosophical explanations do not necessarily reflect those used by individuals (Lombrozo and Vasilyeva, 2017). Explanatory statements used in our daily lives often consist of a small number of features that lack strong casual connections with the underlying phenomenon (cf. Dunbar, 2001). Two *minimal* features that all explanations require include a proposition representing some prior knowledge concerning observed phenomena (e.g., flight, sadness) and a set of explanatory statements that provide accounts of these phenomena (e.g., wing flaps, a failure to achieve a goal). Rather than making assumptions about participants’ prior beliefs about natural phenomena, specific features of explanatory schemata should be identified.

A common feature of explanations (e.g., Murphy, 2000; Keil, 2006; Halford et al., 2010; Wellman, 2011; Lombrozo, 2012) and their application in abductive reasoning in science (Nersessian, 1999; Haig, 2005; Trout, 2007, 2008) is the belief in causal relationships. If there are numerous kinds of explanatory schemata that can be associated with any given class of natural phenomena, the extent to which one is activated and used should depend on the association between features of an explanandum (a phenomenon that needs to be explained), the explanans (the statements explaining the phenomenon) provided by a source outside the individual, as well as the activation of these features and propositions in long-term memory. For instance, previous studies have observed explanatory coherence and simplicity are crucial determinants of the believability of explanations (e.g., Koehler, 1991; Sloman, 1994; Lombrozo, 2007; Douven and Schupbach, 2015; Pacer and Lombrozo, 2017). Although there might be common features of a good explanation, what qualifies as a coherent explanation will likely depend on an understanding domain-specific knowledge.

Participants likely have access to more than one coherent explanatory framework (i.e., explanatory pluralism Dennett, 1987; Kelemen, 1999; Colombo, 2017), can maintain multiple attitudes (Wison et al., 2000), and can acquire a complex schema with minimal exposure to information (Ahn et al., 1992). Participants therefore likely maintain alternative explanatory schemata for any given natural phenomenon defined by multiple features. Explanations can also be understood in terms of analogical reasoning (Gentner et al., 2001; Hobeika et al., 2016), wherein prior knowledge of relational structures can facilitate problem-solving (e.g., Rumelhart and Norman, 1981; Gick and Holyoak, 1983; Vosniadou and Ortony, 1989; Dunbar, 2001). Despite the benefits of prior knowledge, we argue that the extent to which it influences judgment and decision-making is dependent on its compatibility with current task demands which will affect accuracy and subjective confidence. In that coherence has been considered an important feature of explanation (Sloman, 1994; Pacer and Lombrozo, 2017), coherent explanations from one domain might create interference in another domain if there are superficially similar elements in both schemata (for evidence in children, see Richland et al., 2010).

Evidence for such compatibility effects can be found in the literature on reasoning. A number of cognitive factors can bias decision-making for or against the acceptance of an argument (for a review, see Gilovich et al., 2002). For instance, novice participants tend to ignore the logical structure of an argument (i.e., its validity) and instead focus on the believability of its conclusions (i.e., the belief-bias effect; Evans et al., 1983; Markovits and Nantel, 1989; Newstead and Evans, 1993; Sa et al., 1999; Klauer et al., 2000; Stanovich and West, 2000; Evans and Curtis-Holmes, 2005; Trippas et al., 2017). In Evans et al.’s (1983) study, participants were presented with syllogisms that contained a conclusion that is contrary to social representations of smoking:

Premise 1: All things that are smoked are good for the health.
Premise 2: Cigarettes are smoked.
Conclusion:  Cigarettes are good for your health.

In this case, while participants are likely aware that smoking is associated with health risks, they cannot disregard this prior belief and solely focus on the validity of the argument. Within a given domain, participants likely maintain prior beliefs about causal relationships between explanans and explanandum. These prior beliefs might act as heuristics that interfere with judgments of the validity of a conclusion (Newman et al., 2017; Trippas et al., 2017). For instance, in a recent categorization study, Schoenherr and Lacroix (2020) found that exemplars from categories associated with prior knowledge that was unrelated to the category structure interfered with correct categorization but increased subjective confidence. Consequently, we must consider specific domains that might influence judgments of validity and perceptions of certainty.

A persistent question in the study of explanations concerns whether there are distinct *kinds* of explanations that correspond to domain-specific knowledge. Naive and folkscientific theories have been examined in philosophy (Dennett, 1987; Dacey, 2017), developmental psychology (Carey, 1985; Waxman et al., 2007), and cognitive science (e.g., Caramazza et al., 1981). For instance, developmental studies have found that children make conceptual distinctions between “living” and “non-living” entities (e.g., Carey, 1985; Miller and Aloise, 1989; Mandler and McDonough, 1993, 1998) and ascribe qualitatively different intentional responses for objects that apparently exhibit biological motion (for reviews see Poulin-Dubois, 1999; Johnson, 2000; Wellman et al., 2001) and have a basic understanding of goal-directed behaviors (e.g., Poling and Evans, 2002; Goldberg and Thompson-Schill, 2009). In Western society, in addition to developing folk theories concerning physics (e.g., Caramazza et al., 1981; Hubbard and Ruppel, 2013), people are taught to conceive of the world in terms of cause and effect with objects interacting with one another via abstract forces (e.g., Nisbett, 2003; Wolff, 2007). A question that has been left relatively unexamined outside of science educational studies (e.g., Bartov, 1981; Tamir and Zohar, 1991; Talanquer, 2007, 2010; Bardapurkar, 2008; Thulin and Pramling, 2009; Barnes et al., 2017) concerns whether different kinds of explanations facilitate communication, learning, and decision-making within a specific context.

#### Scientific Explanations and Scientific Communication

In that scientific communications can rely on domain-specific content knowledge (e.g., subatomic particles and other unobservable forces) and can pertain to domains that participants are likely to have strong beliefs about (e.g., whether the world is deterministic, and whether all animals think and feel in the same manner as humans; e.g., Hirschfield and Gelman, 1994; Medin and Atran, 1999; Brem and Rips, 2000; Atran and Medin, 2008), prior beliefs will exert considerable influence on performance in decision-making and reasoning tasks (e.g., for examples in studies of folk theories of physics, see Caramazza et al., 1981; Chi et al., 1981; McCloskey, 1983; McCloskey and Kohl, 1983; Proffitt and Gilden, 1989; McAfee and Proffitt, 1991). Supporting this, a meta-analysis conducted by Johnson and Eagly (1989) found that the importance of a belief is inversely related to attitude change. Concurrently, studies of health-beliefs also suggest that specific features of explanatory schemata (e.g., benefits of treatment, disease severity) varied in terms of their relationship with compliance behaviors (Carpenter, 2010). Consequently, features of explanatory schemata will be associated with stronger or weaker beliefs.

Models of attitude change have additionally suggested that subjective confidence can be used to determine the amount of processing that an individual will perform when provided with novel information (Chen and Chaiken, 1999; Chaiken and Ledgerwood, 2012). Initially, a participant sets a criterion for the desired level of confidence (e.g., 80%) and monitors the obtained confidence in the information they have available (e.g., 50%) to determine the amount of effortful processing that is required to increase subjective confidence to the desired level (Chaiken, 1980). Indeed, studies of attitudinal certainty indicate that confidence can be increased by the amount of consideration, direct experience, and the limited diversity of counter-attitudinal evidence (for a review, see Glasman and Albarracín, 2006). When the subject of scientific explanation is related to familiar folk theories, we would therefore expect participants to disregard general reasoning strategies and focus on familiar content contained within explanatory schemata that would lead to higher levels of subjective confidence (Scharrer et al., 2012). Thus, the features of the explanations themselves and how they correspond to prior beliefs contained within explanatory schemata, must be accounted for when considering the effectiveness of scientific explanations and perception of certainty in an explanation.

Recently, a number of studies have provided some evidence for various features of scientific explanations that are relevant to our understanding of psychosocial phenomena. Weisberg et al. (2008) investigated the acceptability of mechanistic explanations of psychological phenomena. In their study, participants were presented with either ‘good’ (non-circular) or ‘bad’ (circular) explanations of human behavior, conforming to elementary psychological phenomena found in undergraduate psychology textbooks. These explanations were either provided alone or were accompanied by irrelevant neuroscientific evidence. They found that novices (second year cognitive neuroscience students) were more likely to find such explanations believable when accompanied by the irrelevant information, relative to experts who were more likely to ignore irrelevant information (see also, Rhodes et al., 2014). Similarly, McCabe and Castel (2008) found that neuroscientific imagery (e.g., brain scans) had a greater effect than other representations (e.g., bar graphs or abstract maps) on the acceptance of explanations. Krull and Silvera (2013) have provided further supporting evidence that the association between the explanans and a scientific instrument (e.g., an MRI) affects the believability of the statement. Taken together, these results suggest that prior beliefs are a significant determinant of participants’ responses and that the pervasiveness of neuroscientific explanations in the media (Beck, 2010; O’Connor et al., 2012) might reflect a central feature of participants’ naïve theories of psychosocial phenomena (cf. Hook and Farah, 2013; Michael et al., 2013).

Prior to accepting these promising conclusions, the properties of these experimental paradigms should be considered as they might prohibit a straightforward interpretation of these results. First, studies have used images or concepts that have concrete images associated with them influence processing. For instance, studies have demonstrated that words invoking vivid imagery are more persuasive (Rossiter and Percy, 1978; Burns et al., 1993; Schlosser, 2003). After controlling for participants’ prior beliefs about the results of a fictitious study (e.g., the effect of music on studying), Rhodes et al. (2014) replicated the Weisberg et al.’s (2008) finding that neuroscientific evidence influenced believability of an explanation of psychological phenomena. However, it might be that the vividness of the real (or invoked) imagery of the brain might be the principal influence on judgments of believability. Such results might not be as informative about critical features of scientific explanations as they are about a specific kind of scientific explanation: a conjunction of imagery and tacit beliefs associated with an argument. Supporting this, a large-scale study conducted by Michael et al. (2013) attempted to replicate the influence of imagery (McCabe and Castel, 2008) using multiple methods of presentation (online and written), multiple participant pools (general public, MTurk, and undergraduates) as well as multiple incentives (e.g., none, course credit, and financial compensation). They failed to replicate previous results (see also, Hook and Farah, 2013). We believe that these observations support a more conservative approach to the study of scientific explanations. Rather than assuming that neuroscientific evidence is itself primary, specific heuristics should be identified. Moreover, accuracy and certainty in judgment must be assessed independently in order to differentiate between the effects of specific heuristics and the strength of their influence on judgments.

### Present Research: Explanatory Schemata in Scientific Reasoning

The present study seeks to examine what features of scientific explanations affect the accuracy of assessing an explanation’s validity and response confidence. We assume that scientific explanatory schemata can be reduced to a finite set of features that can be associated with prior knowledge. Rather than presenting explanations along with neuroscientific evidence (e.g., Weisberg et al., 2008; Rhodes et al., 2014), we used a contrasting set of explanandum (animate and inanimate natural phenomena) and explanans (intentional and mechanistic) to determine whether these features were integrated into a schema or represented distinct heuristics.

#### Essential Features of Scientific Explanation

An issue with previous studies of scientific explanation is that neuroscientific “evidence” such as fMRI scans might be confounded with the source of this evidence, i.e., credible neuroscientists and neuroscience paradigms. Studies of persuasive communication (Wilson and Sherrell, 1993; Petty et al., 1997; Chaiken and Trope, 1999; Sparks and Rapp, 2011) have demonstrated that features of a message such as expertise (Rhine and Severance, 1970; Pornpitakpan, 2004) and source trustworthiness (Mills and Jellison, 1967; Petty et al., 1999) can influence attitudes toward a message (Eagly and Chaiken, 2005; Crano and Prislin, 2006). When the source is deemed to be incompatible with a message, significant reductions in perceived message credibility can be observed (e.g., Petty and Cacioppo, 1986; Hitt et al., 2016). Experts are not the only source of information. In some situations, collective knowledge might be deemed credible, with social proof being a widely used heuristic to make judgments (e.g., Goethals and Darley, 1977; Cialdini, 1984/2007; Miller, 1984; Cialdini and Goldstein, 2004). In the present study, we control for source by comparing explanations offered by “people” to those offered by “scientists.”

Across two experiments we sought to examine which features of scientific explanatory schemata are used to judge the perceived validity of arguments regarding natural phenomena. Following from studies of the belief-bias effect, we assume that participants’ accuracy in a reasoning task will be influenced by their prior beliefs about natural phenomena (Evans et al., 1983; Sa et al., 1999). When the contents of syllogisms are consistent with a prior belief, participants are more likely to judge a syllogism as valid in comparison to when the contents are inconsistent (Oakhill et al., 1989). Thus, if participants believe that mechanistic explanations of natural phenomena are more appropriate than intentional explanations, their accuracy will be greater in the logically consistent condition for mechanical explanations than for intentional explanations. Moreover, we assume that argument validity should affect response accuracy even when participants have received training in syllogistic reasoning (Johnson-Laird and Steedman, 1978; Johnson-Laird and Byrne, 2002). Following from studies of scientific explanation (Weisberg et al., 2008; Rhodes et al., 2014), we additionally assume that participants will tend to perceive mechanistic explanans as valid even when the explanans is inconsistent with the explanandum. While intentional explanans might be more intelligible to participants (Bartov, 1981) due to the use of high frequency terms (i.e., “like,” “wants”), their limited linguistic complexity (Bradac et al., 1977) in contrast to mechanistic terms will make these explanans appear to be less valid even when the explanans is consistent with the explanandum. We assume that participants will prefer compatible explanans-explanandum as they are associated with a pre-existing heuristics or schemata in memory. In the context of the present study, this was operationalized in terms of animate-intentional and inanimate-mechanistic explanatory schemata.

For these same reasons, we also assume that explanans and explanandum tend to be associated with specific sources. Expertise is relative to a domain (e.g., French et al., 2011) and participants likely maintain folk theories about what scientists and people know. Specifically, scientists are more likely to be associated with valid explanans of inanimate explanandum (e.g., physical and chemical processes) whereas people are more likely to have valid knowledge in terms of folkwisdom of animate explanandum (e.g., goal-direct behavior). Priming participants with features of a schema (when available) will therefore increase the likelihood that it will affect response selection. However, following studies of persuasive communication (e.g., Pornpitakpan, 2004), we additionally assume that the importance of source credibility might diminish when manipulating the explanans and explanandum due to participants domain-specific beliefs about source credibility and animacy.

#### Explanation and Certainty

One feature that has yet to be addressed in previous research on scientific explanations is whether participants are aware of their response biases. In general, metacognitive studies of knowledge assessment suggest that individuals overestimate their knowledge (Lichtenstein and Fischhoff, 1977; Kruger and Dunning, 1999; Paulhus et al., 2003). This might be a consequence of using availability and familiarity as a heuristic (Kahneman and Tversky, 1972; Tversky and Kahneman, 1973). For instance, Keil and Wilson (2001) observed that “most common explanations… have a structure that is more *implicit* and schematic in nature than is suggested by more traditional psychological accounts,” (p. 12; italics added). Similar observations have been made in the reasoning literature (e.g., Newman et al., 2017). Consequently, implicit knowledge might lead to discrepancies between response accuracy and response confidence (Ziori and Dienes, 2008; Schoenherr and Lacroix, 2020).

Models of persuasive communication have also considered this possibility. Namely, Chaiken’s (1980; Chen and Chaiken, 1999; Chaiken and Ledgerwood, 2012) Heuristic-Systematic Model of persuasive communication suggests that subjective confidence plays an integral role in determining how much information is gathered: as the desired level of confidence increases, the amount of information processing required also increases. However, studies of reasoning have demonstrated little correspondence between subjective confidence and response accuracy (e.g., Shynkaruk and Thompson, 2006). Indeed, using observation from the belief perseverance literature, Koehler (1991) suggests that the availability of a prior explanation might result in overconfidence. Providing some evidence to support this, Lombrozo (2007) found that simpler explanations are more probable than more complex explanations, even when the more complex explanation has a higher probability of being accurate. However, given that the probabilistic information used to inform a decision and subjective probabilities used to report certainty need not be equivalent, the relationship between beliefs and overconfidence in explanations needs to be directly examined.

Insight into the relationship between performance in assessing the validity of explanation and confidence can be gained by considering studies of trust in science (Kraft et al., 2015; Achterberg et al., 2017). In their study of layperson evaluation of scientific information, Scharrer et al. (2012) found that participants were more likely to trust their own decisions and were less inclined to indicate that they needed the assistance of an expert when information was easy to comprehend. Moreover, in a study conducted by Achterberg et al. (2017) they found respondents reported greater trust for scientific methods relative to scientific institutions, with this discrepancy increased with lower levels of scientific literacy. However, these studies were not concerned with the extent to which these judgments were well calibrated, i.e., whether subjective confidence corresponded to the accuracy of participants’ knowledge. For instance, studies of general knowledge have observed greater overconfidence relative to perceptual tasks (Kvidera and Koutstaal, 2008) with that difficult questions associated with the greatest levels of overconfidence (Lichtenstein and Fischhoff, 1977). Consequently, research suggests that information that is more accessible in memory (Tversky and Kahneman, 1973; Schwarz and Vaughn, 2002) such as prior knowledge (Kahneman and Tversky, 1972; Koriat and Ma’ayan, 2005; Schoenherr and Lacroix, 2020) can increase response confidence independently of response accuracy.

In line with previous studies, we predict that participants will be miscalibrated in assessing their own judgments. Specifically, explanatory heuristics and schemata related to source credibility (expert and non-expert) relative to specific explanans (inanimate and animate domains) and kinds of explanandum (e.g., mechanistic and intentional) will be associated with greater confidence. We will examine overconfidence bias as it has been demonstrated to be a superior measure of subjective awareness than other metaknowledge measures in that it indicates the participants level of certainty after controlling for performance (Schoenherr and Lacroix, 2020; cf. Ziori and Dienes, 2008). More specifically, we assume that when features of an explanation are associated within a schema (i.e., congruent), we should observe greater overconfidence than when they are not associated (i.e., incongruent).

To assess these predictions, two experiments provided participants with explanans (intentional or mechanistic), explanandum (animate/living or inanimate/non-living), and varied the source of the information (people or scientists). All experiments used the same stimuli described in Experiment 1 below (for examples, see Table 1). In addition to validity judgments, participants were additionally required to rate their confidence. In Experiment 1, we investigated whether mechanistic explanations might provide a more general basis for participants’ acceptance of scientific explanations than in previous studies. In Experiment 2, we examined whether working memory capacity was associated with the extent to which features of an explanation influenced participants’ responses. Moreover, by examining overconfidence bias, we additionally assumed that we can identify specific features of explanatory schemata that were most important to participants.

**TABLE 1:** Samples of syllogisms modified with mechanistic and intentional explanations.

Condition	Example
**Mechanistic**	**Description**: If [a Baje moves toward a Yulo then they will stick together]^P1^
**Consistent**	**Explanations**: [A Baje moves toward a Yulo]^*P2*^ because [Bajes and Yulos are bound by a force]^*E*^ that [attracts them]^*C*^.
**Intentional**	**Description**: If [a Lozu moves toward a Hexi then they will stick together]^P1^
**Consistent**	**Explanations**: [A Lozu moves toward a Hexi]^P2^ because [Lozus and Hexis like one another]^*E*^ so they [are drawn together]^C^.
**Mechanistic**	**Description**: If [a Dafe moves toward a Noha then they will stick together]^*P1*^
**Inconsistent**	**Explanations**: [A Dafe moves toward a Noha]^P2^ because [Dafes and Nohas are bound by a force]^*E*^ that [repels them]^C^.
**Intentional**	**Description**: If [a Vipo moves toward a Pova then they will stick together]^P1^
**Inconsistent**	**Explanations**: [A Vipo moves toward a Pova]^*P2*^ because [Vipos and Povas dislike one another]^*E*^ so they [are driven apart]^*C*^.

*Major and minor premises are denoted by P1 and P2, respectively, and the conclusion is denoted by C. The irrelevant explanation is denoted by E.*

## Experiment 1

One concern with previous studies of scientific reasoning (e.g., Weisberg et al., 2008) is that it was not clear whether both the source of information and the kinds of evidence interacted, suggesting that participants have access to an explanatory schema. For instance, it is reasonable to assume that neuroscientists were involved in the production of neuroscientific evidence rather than a layperson. We sought to examine the conditions in which the source of evidence presented in the context of a scientific explanation influenced participants’ acceptance of explanans and explanandum. In Experiment 1, we explicitly manipulated the source of evidence to examine whether the compatibility between source of information and kind of evidence influences participants’ judgments of syllogisms, such that participants were biased to accept consistent syllogisms when the source of the explanations and the kind of phenomena are compatible and biased to reject consistent syllogisms when the source of the explanations and the kind of phenomena are incompatible.

### Method

#### Participants

A random convenience sample of ninety-five participants from Carleton University received 1% toward their final grade in introductory psychology courses.

#### Materials

Sixteen training syllogisms consisted of modus ponens (MP), modus tollens (MT), hypothetical (HS), and disjunctive (DS). In order to avoid bias associated with content knowledge, syllogism used CVCV (consonant-vowel-consonant-vowel; e.g., Baje, Yulo) non-words as the subjects in the major and minor premises. Syllogisms were presented in a standard format. For instance, a modus ponens was presented with the major premises, followed by the two minor premises:

If there is a Sohi then there is a Loze,
There is a Sohi,
Therefore, there is a Loze

Thirty-two experimental syllogisms used the same format as standard syllogisms with the exception of including a “Description” and an “Explanation.” Descriptions of phenomena contained the major premise in the syllogism (e.g., If a [*Baje moves toward a Yulo*]^*P*^ then [*they will stick together*]^*Q*^). Explanations contained the minor premise embedded in an irrelevant explanatory feature consisting of an intentional (e.g., Ps like each other; [*Bajes and Yulos like one another*]) or mechanistic explanation (e.g., Ps are drawn by a force; [*Bajes and Yulos are bound by a force*]). Finally there is an explanatory feature that is either consistent or inconsistent [*that attracts (repels) them*] with the major premise [*they will stick together*].^*Q*^

Each modified syllogism type (MP, MT, HS, DS) varied in terms of the explanation type (mechanistic or intentional) and validity (valid or invalid). Two versions of each syllogism were created in order to have two replications per condition. For the purposes of syllogistic reasoning, we assumed that the consistent (or inconsistent) explanation was perceived to reflect the implied conclusion Q (or ∼Q). For examples of these stimuli, see Table 1.^1^ In this way, our method is similar to studies that use bad (circular) and good (non-circular) explanations (Weisberg et al., 2008). As we note below, participants’ responses support our assertion that a syllogism’s logical validity affects perceived validity.

#### Procedure

Following the informed consent, and a colloquial explanation of what constitutes logical validity, participants were then provided with the training syllogism session. The 16 training syllogisms were presented to participants for practice and no feedback was provided prior to the start of the experimental session.

Prior to the start of an experimental block, the kind of natural phenomena and source of the explanation were manipulated randomly via an instructional manipulation. Half of the participants were informed that the natural phenomena were “inanimate” (or “non-living”) and the remaining half were informed that the natural phenomena were “animate” (or “living”).

In one experimental block, participants were told that *scientists* observed phenomena and offered explanations, and in the other block participants were told that *people* observed phenomena and offered explanations. The same 32 modified syllogisms were used in both blocks. The order of the scientists or people block manipulation was counterbalanced to avoid order effects.

### Results and Discussion

The experimental design for each of the independent variables used a mixed repeated-measures analysis of variance (ANOVA) consisted of 2 (Intentional or Mechanistic) × 2 (People or Scientists) × 2 (Consistent or Inconsistent) as the within-subject variables. Animacy (living or non-living) and explanandum Formality (formal or informal) were included as between-subjects measures. Instructional order of ‘scientists’ or ‘people,’ syllogism type, and the two replications, were collapsed for the purposes of analyses as these variables were not of interest to the present study.

Participants were included on the basis that they performed above chance (i.e., p(correct) > 0.5). This resulted in the removal of six participants, leaving a comparable number of participants in non-living (*n* = 17) and living (*n* = 16) conditions using informal terms, and the inanimate (*n* = 29) and animate (*n* = 27) conditions using formal terms. However, due to an absence of differences between the formal and informal terminology in a preliminary analysis, we collapsed across these conditions.

Training blocks were excluded from the analysis as the training results from this and subsequent studies did not bare directly on the hypotheses.

We used Greenhouse–Geisser adjusted statistics but report the unadjusted degrees of freedom. The analysis was conducted using SPSS.

#### Response Accuracy

**FIGURE 1:**
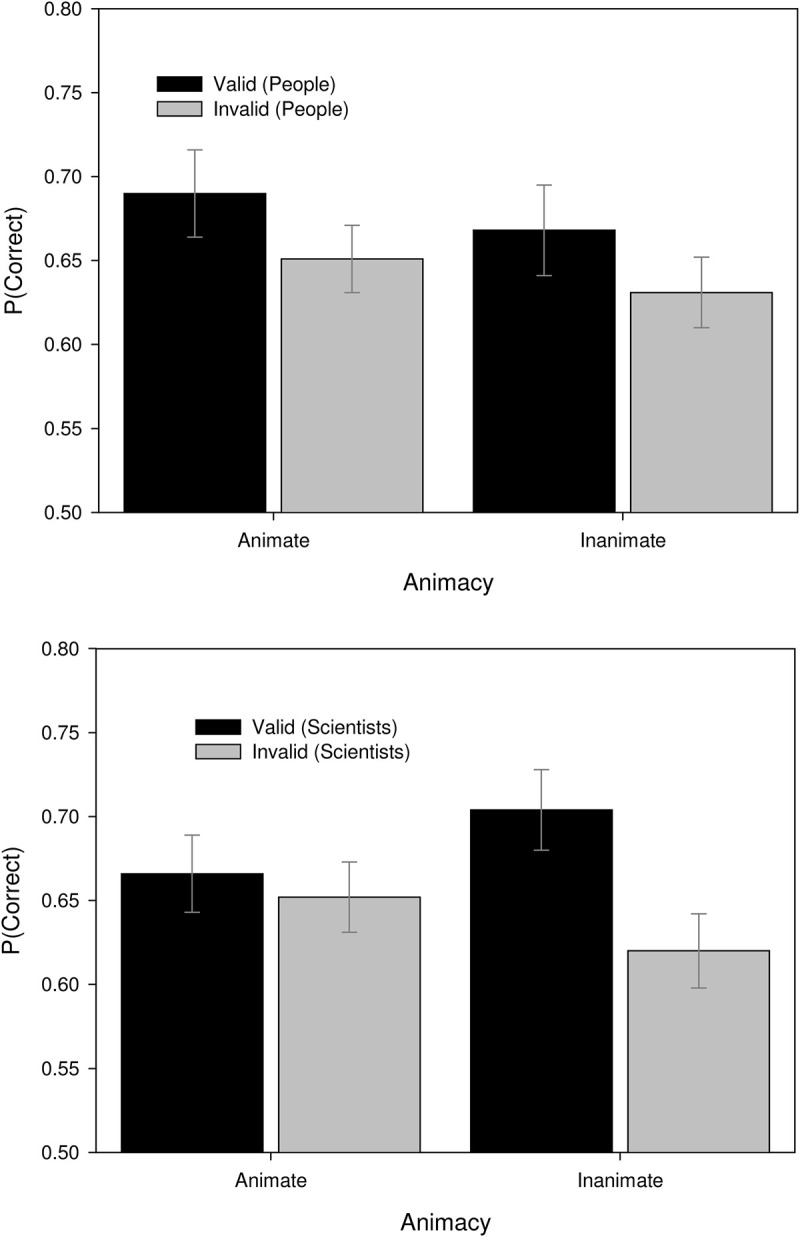
Proportion correct for explanations of animate and inanimate provided by people **(top)** and scientists **(bottom)**. Error bars represent standard error of the mean.

The mixed ANOVA of proportion correct revealed a marginally significant three-way interaction between Source, Animacy, and Validity, *F*(1,85) = 3.65, *MSE* = 0.014, *p* = 0.059, η^2^ = 0.04, ω^2^ = 0.47. As Figure 1 demonstrates, participants believed that people were more likely to provide valid explanations of animate phenomena whereas scientists were more likely to provide valid explanations of inanimate phenomena, thereby producing higher accuracy in these conditions. This suggests specific domains of competency that are associated with the social categories of “people” and “scientists.” This can be understood in terms of the persuasive communication heuristics of social proof (e.g., Goethals and Darley, 1977; Cialdini, 1984/2007; Miller, 1984) and expertise (e.g., Rhine and Severance, 1970; Newhagen and Nass, 1988; Slater and Rouner, 1996; cf. McGinnes and Ward, 1980). Consequently, when a given source is providing an explanation outside of their domain, participants are less likely to believe that it will be valid regardless of the objective validity of the explanation.

The three-way interaction also qualifies a number of main effects and interactions. We obtained a significant interaction between Explanation Type and Validity, *F*(1,85) = 159.16, *MSE* = 0.033, *p* < 0.001, η^2^ = 0.65, ω^2^ = 1.0. Mechanistic explanations were believed to be valid, resulting in high accuracy for consistent syllogisms and chance performance for invalid syllogisms, a finding that replicates research on the effects of argument validity (Evans et al., 1983; Stanovich, 2003). Intentional explanations were generally believed to be invalid resulting in higher accuracy in the inconsistent condition. In contrast, participants exhibited above chance performance when presented with consistent intentional syllogisms. These results suggest either that participants typically considered intentional explanans to be invalid or that mechanistic explanations were typically considered to be valid (see Table 2). A marginal effect of Explanation Type and Animacy was also observed, *F*(1,85) = 3.16, *MSE* = 0.012, *p* = 0.079, η^2^ = 0.04, ω^2^ = 0.42. We found that accuracy was roughly equivalent for both intentional (*M* = 0.65, *SE* = 0.011) or mechanistic explanations (*M* = 0.66, *SE* = 0.012) of animate phenomena, whereas accuracy was greater for mechanistic (*M* = 0.69, *SE* = 0.012) relative to intentional explanations (*M* = 0.64, *SE* = 0.011) of inanimate phenomena. Consequently, participants appear to hold the strongest beliefs for mechanistic explanations of inanimate phenomena.

**TABLE 2:** Overconfidence (OC) for explanation type and consistency.

		Explanation type	
		
		Mechanistic	Intentional
OC	Consistent	0.050 (0.034)	0.195 (0.042)
	Inconsistent	0.310 (0.034)	0.103 (0.031)
	*Mean*	0.180 (0.026)	0.149 (0.025)

*Standard error reported in parentheses.*

Our analysis also revealed a significant main effect of Explanation Type, *F*(1,85) = 8.84, *MSE* = 0.012, *p* = 0.004, η^2^ = 0.09, ω^2^ = 0.84, and a marginal effect of Validity, *F*(1,85) = 2.69, *MSE* = 0.118, *p* = 0.105, η^2^ = 0.03, ω^2^ = 0.37. Thus, block-level manipulations did not have a large effect on performance whereas trial-to-trial information concerning the explanandum and its validity contributed to performance. Crucially, we did not find any significant main effects or interactions of formal or informal terminology (all *p*s ≥ 0.356). Consequently, it appears that participants were using concepts of animate and inanimate and were not influenced by the specific terminology used to describe these concepts.

#### Overconfidence Bias

In order to assess participants certainty, we assessed their response confidence. However, given that confidence and accuracy have a complex relationship (Keren, 1991; Koriat and Ma’ayan, 2005), we decided to control for the effects of accuracy by using a measure of *overconfidence bias*. Participants’ accuracy was subtracted from their reported subjective probability in each condition (e.g., Baranski and Petrusic, 1994). Formally, overconfidence bias is given by the equation:

OCB = (Mean Confidence/100) − Proportion Correct

Thus, OCB represents the extent to which a participant’s mean confidence exceeded their mean accuracy in an experimental condition. The obtained difference score was then included in as a dependent variable in a repeated-measures ANOVA of OCB, which had an identical factorial structure to that of accuracy.

Our repeated-measures ANOVA of OCB replicates the effect of accuracy. We again obtained a significant interaction between Explanation Type and Validity, *F*(1,85) = 177.87, *MSE* = 0.032, *p* < 0.001, η^2^ = 0.68, η^2^ = 0.68, ω^2^ = 1.0. The main effects of Explanation Type, *F*(1,85) = 5.88, *MSE* = 0.014, *p* = 0.017, η^2^ = 0.07, ω^2^ = 0.67 and Validity were also significant, *F*(1,85) = 4.74, *MSE* = 0.113, *p* = 0.032, η^2^ = 0.05, ω^2^ = 0.58.

As Table 2 demonstrates, we observed considerable overconfidence in the valid intentional explanation and the invalid mechanistic explanation conditions. Given that OCB controls for a participant’s accuracy within a given experimental condition (i.e., by subtracting proportion correct), it reflects the strength of the beliefs a participant maintains. Thus, not only did these explanatory biases increase or decrease accuracy, they had an independent effect on participants’ certainty. Moreover, given that confidence reflects an explicit measure of subjective awareness, participants clearly overestimated their accuracy within these domains. The absence of a main effect of explanans terminology (e.g., living or animate) or its interaction with other factors also suggests that the explanans is of greater importance than the terminology used to describe it.

### Discussion

Across measures of accuracy and overconfidence bias, the results of Experiment 1 demonstrate a belief-bias effect: logical consistency and explanans interacted. This can be understood in terms of a heuristic in that participants are influenced by their beliefs about mechanistic and intentional explanans. This qualifies studies that report the effects of explanatory coherence (Lombrozo, 2007; Pacer and Lombrozo, 2017) in that coherence is relative to a domain. The significant effect of explanandum (intentional or mechanistic) suggests that participants generally attend to these features. Thus, folk theories related to intentional and mechanistic phenomena (e.g., Poling and Evans, 2002; Nisbett, 2003; Wolff, 2007; Goldberg and Thompson-Schill, 2009) likely continue to exert an influence on adult reasoning in these domains. While marginal, we also obtained evidence that participants might maintain complex schemata that are influencing their judgments. Specifically, scientists appear to be more credible sources of explanations of inanimate phenomena whereas people appear to be more credible sources of explanations of animate phenomena. Similarly, we also found that participants believe that mechanistic explanans provide a better account of inanimate phenomena relative to animate phenomena. Consequently, participants appear to hold the strongest beliefs for mechanistic explanations of inanimate phenomena.

While response accuracy and overconfidence were only influenced by explanans, our analysis of accuracy found evidence that participants’ performance was influenced by more complex associations such as those between the source of an explanans, the animacy of the explanandum, and the validity of the explanation. These results suggest that participants likely maintain a schema wherein scientists are reliable sources of information of inanimate phenomenon but laypersons can provide valid explanations of animate phenomenon.

## Experiment 2

Experiment 2 sought to extend the results of Experiment 1 by decreasing the likelihood that participants could use executive functioning during the reasoning task. Previous research has indicated that the role of working memory is central to performance in syllogistic reasoning tasks (Stanovich and West, 2008). Namely, reasoning is believed to be associated with an effortful processing rather than automatic biases (Evans et al., 1983). In Experiment 1, a failure to observe the effects of source or animacy in the analysis of overconfidence might be attributable to participants’ limited cognitive resources. Consequently, in Experiment 2, we measured each participant’s working memory capacity to include as a between-subjects variable, as studies have demonstrated a positive relationship with task performance (e.g., Kyllonen and Christal, 1990; Süβ et al., 2002). We also used a concurrent load to increase the likelihood that participants would rely on heuristics related to the explanandum and explanans rather than engage in effortful reasoning.

### Methods

#### Participants

Forty-seven participants from Carleton University performed the task for 1% course credit.

#### Materials and Procedure

Prior to the start of the experiment, participants were provided with a brief working memory task. Working memory stimuli for the letter span tasks consisted of 10 random letter strings, each containing eight letters. Their responses to the memory task were then used to separate participants into high- and low-working memory capacity groups for later analysis.

Training syllogisms and modified syllogisms were identical to Experiment 1. In contrast to Experiment 1, Experiment 2 did not manipulate explanation source in the primary task, i.e., people or scientists.

Prior to each trial, participants were provided with a concurrent load consisting 1 of 32 unique random number strings. Each string was presented for 1 s prior to receiving the syllogism and consisted of four random letters. Participants recorded the recalled items following their validity judgment and subjective confidence report. Thus, the load persisted both for the primary decision and confidence report phases of the experiment.

### Results

Rather than using a median split to differentiate participants based on working memory capacity, we used a theoretically important distinction. Specifically, using Cowan’s (2001) suggestion that the capacity of working memory when chunking was inhibited is four items, the results of the working memory task were used to create a high-capacity group (WM_*score*_ > 4) and a low-capacity group (WM_*score*_ < 4). The high-capacity criterion resulted in slight differences in the number of participants in the high-capacity group (*n* = 21) relative to the low-capacity group (*n* = 25). Participants were included on the basis of the performance criterion such that they needed to perform above chance, i.e., *p*(corr) > 0.5. This resulted in the removal of ten participants who appeared to be guessing throughout the task. The sample remained equivalent for low- (*n* = 18) and high-working memory capacity (*n* = 19) groups. One additional participant in the high-working memory capacity condition was removed from the analysis of overconfidence due to an error in reporting response confidence.

#### Response Accuracy

Replicating Experiment 1, an analysis of response accuracy revealed an interaction between Explanation Type and Validity, *F*(1,33) = 41.37, *MSE* = 0.026, *p* < 0.001, η^2^ = 0.56, ω^2^ = 1.0. We also did not find a significant effect of Animacy, *F*(1,33) = 0.805, *MSE* = 0.020, *p* = 0.38, η^2^ = 0.024, ω^2^ = 0.141. Moreover, we only obtained a marginal effect of Validity, *F*(1,33) = 2.98, *MSE* = 0.054, *p* = 0.094, η^2^ = 0.083, ω^2^ = 0.39, and no effect of Working Memory Capacity Group, *F*(1,33) = 0.005, *MSE* = 0.020, *p* = 0.946, η^2^ = 0.000, ω^2^ = 0.05. The absence of an effect might appear odd given the role of working memory in reasoning tasks (Stanovich and West, 2008). Concurrently, the low observed power might suggest that a much larger sample size is required in order to account for individual differences. However, when compared to Experiment 1, working memory load appears to have reduced accuracy uniformly across explanatory conditions. Given that the letter string we provided to participants were based on average working memory capacity (Cowan, 2001), individual differences in working memory capacity might only provide a negligible advantage.

Consistent with Experiment 1 (see Table 2), participants exhibited relatively higher accuracy judging valid mechanistic explanations compared to invalid mechanistic explanations. Intentional explanations again produced the opposite pattern of performance, with participants instead exhibiting relatively higher accuracy for inconsistent intentional explanations compared to consistent intentional explanations.

#### Overconfidence Bias

Overconfidence bias was obtained in the same method as Experiment 1. Similar to Experiment 1, we found a significant interaction between Explanation Type and Validity, *F*(1,32) = 41.91, *MSE* = 0.026, *p* < 0.001, η^2^ = 0.57, ω^2^ = 1.0. The greatest level of overconfidence bias was obtained for valid mechanical explanations and invalid intentional explanations (see Table 3). This suggests that participants maintained a strong belief in their responses further implying that they believed mechanistic explanans were generally correct while intentional explanans were generally incorrect.

**TABLE 3:** Proportion correct [P(COR)], mean confidence (CONF), and overconfidence (OC) for explanation type and consistency for Experiment 2.

		Explanation type	
		
		Mechanistic	Intentional
P(COR)	Consistent	0.759 (0.018)	0.606 (0.019)
	Inconsistent	0.537 (0.014)	0.740 (0.017)
CONF	Consistent	83.40 (1.07)	84.47 (1.00)
	Inconsistent	85.48 (0.91)	85.03 (0.93)
OC	Consistent	0.075 (0.020)	0.239 (0.021)
	Inconsistent	0.318 (0.015)	0.110 (0.019)

*Standard deviation reported in parentheses.*

Similar to response accuracy, we again did not find any differences between Working Memory Capacity Groups, *F*(1,32) = 0.70, *p* = 0.41, η^2^ = 0.022, ω^2^ = 0.129, or an effect of Animacy, *F*(1,32) = 0.36, *p* = 0.55, η^2^ = 0.011, ω^2^ = 0.09. Replicating our previous results, we also observed a significant effect of Validity, *F*(1,32) = 4.15, *MSE* = 0.059, *p* = 0.05, η^2^ = 0.12, ω^2^ = 0.51. Thus, even after controlling for participant’s performance and accounting for working memory capacity, overconfidence bias was still affected by the same factors as accuracy.

### Discussion

The results of Experiment 2 replicated those of Experiment 1, suggesting several important features about the explanatory schema activated in this task. First, the belief-bias effect for mechanistic and intentional explanans was observed even with the provision of a concurrent working memory load. Although participants’ working memory capacity (as measured by a letter span task) did not affect the expression of this bias, the addition of a concurrent digit span task to the minimal explanation task did systematically decrease performance. This systematic decrease would seem to suggest that similar processes operated when reasoning about mechanistic and intentional explanans thereby indicating that one explanatory schema was not likely to be more readily available than another. Consequently, what mattered was whether a partial explanans was primed on a given trial.

Replicating the findings of Experiment 1, we observed a bias toward the acceptance of mechanistic explanations relative to intentional explanations. The results of the present task suggests that explanans might be more accessible or relevant than explanandum and, in particular, that participants maintain heuristics that support the acceptance of mechanistic explanations. Alternatively, it might be the case that limitations in working memory make block-level priming of explanandum less effective. Both of these factors appear to contribute to the results of Experiment 2. When compared to previous studies of scientific explanations (e.g., McCabe and Castel, 2008; Weisberg et al., 2008), the results of Experiments 1 and 2 suggest the more general conclusion that mechanistic explanations, rather than neuroscientific explanations in particular, might be the source of participants’ subjective bias.

## General Discussion

Scientific explanations are typically considered in terms that scientists themselves would understand (e.g., Hempel and Oppenheim, 1948; Salmon, 1989; Strevens, 2008). Effective scientific communication (Zimmerman et al., 2001; Bromme et al., 2011; Scharrer et al., 2012; Thomm and Bromme, 2012) and education (Bartov, 1981; Tamir and Zohar, 1991; Talanquer, 2007; Barnes et al., 2017) requires an understanding of the essential features of explanations of natural phenomena (for a discussion of the extent to which explanatory ‘primitives’ exist, see Glennan, 2009). Studies of reasoning (Evans et al., 1983; Markovits and Nantel, 1989; Trippas et al., 2017), decision-making (Tversky and Kahneman, 1973; Schwarz and Vaughn, 2002), and explanation (Lombrozo, 2007) suggest that simple heuristics are likely used to understand many natural phenomena. The availability of multiple simple explanations including folk theories (e.g., Carey, 1985; Waxman et al., 2007; Goldberg and Thompson-Schill, 2009) and abstract forces (Nisbett, 2003; Wolff, 2007) requires that we identify which explanans are associated with specific explanandum in order to understand the effectiveness of the explanation.

To explore this, the current study used a “minimal explanation” paradigm. Using pseudowords that referred to fictional entities, we manipulated explanans (mechanistic and intentional) and explanandum (animate and inanimate phenomena) provided by ‘people’ and ‘scientists.’ In addition to making judgments of the validity of these explanations participants reported their subjective confidence. By examining the accuracy of validity judgments and overconfidence bias, we were able to dissociate participants’ knowledge and their subjective awareness of their performance.

The results of two experiments suggest that minimal explanatory schemata are activated when judging the validity of explanations in a reasoning task. Our findings demonstrate that intentional and mechanistic explanans are associated with differences in the degree of their perceived validity in accounting for specific phenomena, with a tendency to reject the validity of intentional explanations and a tendency to accept the validity of mechanistic explanations. Similarly, the source of a given explanation (scientists vs. people) appeared to be strongly associated with specific explanans (scientists∼mechanistic and people∼intentional), which suggests that participants maintain folk theories about the competencies of members of these social categories. Animate phenomena had similar strong associations with intentional explanations whereas inanimate phenomena were associated with mechanistic explanations. Taken together, both of these results replicated the general finding that believability affects judgements of validity (e.g., Evans et al., 1983; Stanovich and West, 2007). However, rather than these individual heuristics being applied separately (i.e., a main effect), we found evidence that more complex explanatory schemata can be used by participants (i.e., multiple interactions). Supporting this, a recurrent finding across two experiments in the current study is that participants sometimes rely on heuristics (i.e., mechanistic explanations are accurate) whereas at other times associative schemata affect decision-making (e.g., scientists’ explanations of inanimate phenomena are accurate).

### Accessibility of Explanatory Heuristics

A straightforward means to account for the findings of the present study is the broad distinction proposed between effortful, algorithmic, and controlled processing (Type 2 Processes) and effortless, heuristic, and automatic processing (Type 1 Processes) in the cognitive and social-cognitive psychology literatures (e.g., Chen and Chaiken, 1999; Stanovich, 2004; Chaiken and Ledgerwood, 2012). Evidence for the involvement of multiple processes comes from our analysis of response accuracy and response confidence. By controlling for response accuracy, overconfidence bias can be examined to assess participants’ subjective awareness of their own knowledge (Ziori and Dienes, 2008; Schoenherr and Lacroix, 2020). Research in a number of domains has identified discrepancies between people’s knowledge of a domain and their confidence (e.g., Lichtenstein and Fischhoff, 1977; Paulhus et al., 2003; Schoenherr and Lacroix, 2020). Specifically, people tend to be overconfident when they are presented with general knowledge questions (Lichtenstein and Fischhoff, 1977; Paulhus et al., 2003; cf. Kvidera and Koutstaal, 2008), with other studies finding discrepancies between confidence and knowledge within specific domains (Drummond and Fischhoff, 2017; Motta et al., 2018; Park et al., 2020). As Schoenherr and Lacroix (2020) demonstrated, these results appear to be a consequence of the availability of knowledge rather than from the structure and content of knowledge (cf. Ziori and Dienes, 2008). This result conforms to models of performance monitoring that assume that subjective reports such as confidence ratings have access to multiple sources of information (e.g., Koriat and Ma’ayan, 2005; Schoenherr et al., 2010) as well as general heuristics (Tversky and Kahneman, 1973; Schwarz and Vaughn, 2002). As such, participants likely maintain multiple, independent heuristics for explanans and explanandum that can be associated into more complex explanatory schemata through repeated activation. Concurrently, participants likely use the availability of information (Tversky and Kahneman, 1973; Schwarz and Vaughn, 2002; Koriat and Ma’ayan, 2005) or prior knowledge structures in memory as the basis for their judgments of certainty (Ziori and Dienes, 2008; Schoenherr and Lacroix, 2020).

Our overconfidence results also suggest important features of explanations. Supporting other studies of causal features (e.g., Rehder and Burnett, 2005; Rehder and Kim, 2010), our results suggest that explanans occupy a central position in explanatory schemata. Similarly, persuasive communication studies have observed that while the credibility of a message source increases the acceptance of a message when presented alone, when presented along with other information source credibility has a negligible effect (Pornpitakpan, 2004). In contrast, Experiment 1 found that the source of the explanation affected performance while it did not affect overconfidence bias. Thus, participants likely did not have explicit awareness of how these associations affected their performance. Although our results suggest that mechanistic explanans are used to understand psychosocial phenomena (e.g., Weisberg et al., 2008), there is additional evidence for the use of intentional explanations. Taken together, it appears that participants might be generating mental models to solve syllogisms (Johnson-Laird, 1983; Khemlani and Johnson-Laird, 2012), however, such models might not reflect a dense associative network or schemata. Instead, they likely represent minimal explanations.

### The Invalidity of Intentional Explanans

Participants were generally biased toward the *rejection* of intentional explanans and *acceptance* of mechanistic explanans. Specifically, despite the robust finding that response accuracy is higher for valid relative to invalid syllogisms in the syllogistic reasoning literature, we found that response accuracy was nearly equivalent for consistent and inconsistent intentional explanans suggesting a response bias associated with beliefs about intentional explanations. It might be the case that this over-attribution of intentional states is suppressed because mechanistic cues de-emphasize applicability of psychosocial cues and animacy more generally. Given that the stimuli in the minimal explanation paradigm lacked many features that would normally be associated with intentional targets (i.e., reference to motion or an ontological category) only a limited amount of activation is likely to have occurred to support an intentional bias. Furthermore, this activation might have been lost in a competition with processes associated with abstract, mechanistic reasoning.

Supporting this, we did not observe the robust relationship between greater response accuracy in conditions wherein syllogisms were consistent relative to conditions wherein they were inconsistent. Instead, participants’ performance decreased when responding to consistent intentional syllogisms relative to consistent mechanistic syllogisms. Such a bias might then be construed as a specific case of the belief-bias effect (e.g., myside bias; Stanovich and West, 2007). Following from other studies, participants might simply use consistent and inconsistent conclusions to determine believability and validity (Oakhill et al., 1989).

It could also be argued that what we have observed here is a result of a general teleological theory: that goal-directed behavior is in fact the cause of the bias rather than intentionality *per se* (e.g., Poling and Evans, 2002; Goldberg and Thompson-Schill, 2009). However, both the mechanistic and intentional stimuli contained goal-directed premises. Statements such as “A Luze is drawn to a Blix” and “A Luze wants to be near a Blix” could both imply that the end state is that a Luze and Blix *should* come into contact or be in close proximity to one another. Consequently, although a teleological reasoning heuristic might be relevant for these stimuli, our findings suggest that participants likely possessed a specific intentional bias. Additional research could examine whether specific biases exist for teleological explanations, whether they represent an intermediary condition between mechanistic and intentional, or whether they are comparable to either type of explanation.

### Source Credibility Effects in Syllogistic Reasoning

While source credibility has been studied extensive in the attitude and attitude change literature (Cialdini and Goldstein, 2004), it has only been examined relatively recently in the context of a syllogistic reasoning paradigm (Copeland et al., 2011). For instance, in Experiment 2 of Copeland et al. (2011), participants were presented with syllogisms provided by an “expert” (Harvard Psychologist) and a “non-expert” (Mechanic). They found that participants were more likely to accept conclusions that were provided by experts relative to non-experts. While these results need to be replicated (cf. Boucher, 2014), the findings of the present study lend some qualifying support to these conclusions.

Importantly, the effect of the source of the explanation took on a form that is quite different than would have been expected by Copeland et al. (2011). Rather than associating scientists with high credibility, participants were most accurate when scientists were believed to be the source of explanations for inanimate/mechanistic phenomena. In contrast, participants were equally accurate when “scientists” or “people” were thought to be the source of conclusions of animate/intentional phenomena. One possibility is that participants might simply have assumed that scientists and people had differential expertise and that is why the experimenters provided syllogisms from these two sources. Providing support for this, when an analysis only considered formal terms, the stimulus set used in the present study demonstrated associations between scientists and inanimate phenomena as well as people and animate phenomena (for a preliminary analysis, see Schoenherr et al., 2011). These results conform to the literature on attitude and attitude change that demonstrates the influence of both social proof and source credibility (e.g., Goethals and Darley, 1977; Cialdini, 1984/2007; Miller, 1984). Thus, participants in our study might have assumed that “people” as a group possess a satisfactory understanding of animate phenomena whereas “scientists” have an exclusive understanding of inanimate phenomena. In contrast to other studies (Copeland et al., 2011), our study provides evidence for the influence of non-expert opinions when reasoning about judgments of validity. Reasoning strategies such as this might provide the basis for acceptance of complementary and alternative medicines (e.g., Astin, 1998; Kaptchuk and Eisenberg, 1998; Frass et al., 2012; cf. Hitt et al., 2016).

More generally, our research adds to recent studies that examine how extraneous information can influence judgments about the validity of scientific arguments. It is clear from the present study that the effect observed by Weisberg et al. (2008; see also, McCabe and Castel, 2008) is not limited to neuroscientific explanations, and that *many* more factors need to be controlled when examining such reasoning biases. Failures to replicate this effect (Hook and Farah, 2013; Michael et al., 2013) might stem from such factors. In order to develop more effective scientific communications, further research is required to specific the specific features of scientific (e.g., Kraft et al., 2015) and medical explanations (Carpenter, 2010; Park et al., 2020) that make them persuasive. By using a minimal explanation paradigm that differentiates between assessments of validity and certainty, greater insight can be gained into what makes an explanation believable.

## Data Availability Statement

The aggregated data supporting the conclusions of this article will be made available by the authors, without undue reservation.

## Ethics Statement

The studies involving human participants were reviewed and approved by Department of Psychology, Carleton University. The participants provided their written informed consent to participate in this study.

## Author Contributions

JRS conducted the literature review, experimentation, and analysis. JRS and RT collaborated in developing the stimuli and conceptual framework and interpretation. RT provided insight into the requirements of ensuring that the statements conformed as closely as possible to logical syllogisms. Both authors contributed to the article and approved the submitted version.

## Conflict of Interest

The authors declare that the research was conducted in the absence of any commercial or financial relationships that could be construed as a potential conflict of interest.

## Publisher’s Note

All claims expressed in this article are solely those of the authors and do not necessarily represent those of their affiliated organizations, or those of the publisher, the editors and the reviewers. Any product that may be evaluated in this article, or claim that may be made by its manufacturer, is not guaranteed or endorsed by the publisher.

## Footnotes

1 While the use of antonyms (*repels* vs. *attracts*) are not contradictory terms (technically, they are contraries), in the context of our explanations they serve an equivalent purpose (e.g., *repels* denotes a case of *not-attracts*) and thus lead to the same judgment for the explanatory element. While it could be argued that the syllogism is not strictly valid, based on vernacular use of antonyms (supporting ecological validity of the statements; otherwise, they would be grammatically awkward and too similar to strict syllogisms) the perceived validity of the statements is similar to the strict validity had we used contradictory terms rather than contraries. In other words, the truth conditionals of our syllogisms do not change whether we had used contraries (i.e., antonyms) or contradictions.

## References

[B1] Achterberg P. De Koster W. Van der Waal J. (2017). A science confidence gap: education, trust in scientific methods, and trust in scientific institutions in the United States, 2014. *Public Underst. Sci.* 26 704–720. 10.1177/0963662515617367 26644011

[B2] Ahn W.-K. Brewer W. F. Mooney R. J. (1992). Schema acquisition from a single example. *J. Exp. Psychol.* 18 391–412. 10.1037/0278-7393.18.2.391

[B3] Astin J. A. (1998). Why patients use alternative medicine: results of a national study. *JAMA* 279 1548–1553. 10.1001/jama.279.19.1548 9605899

[B4] Atran S. Medin D. (2008). *The Native Mind and the Cultural Construction of Nature.* Cambridge: MIT Press.

[B5] Baranski J. V. Petrusic W. M. (1994). The calibration and resolution of confidence in perceptual judgments. *Percept. Psychophys.* 55 412–428. 10.3758/bf03205299 8036121

[B6] Bardapurkar A. (2008). Do students see the “Selection” in organic evolution? A critical review of the causal structure of student explanations. *Evolution* 1 299–305. 10.1007/s12052-008-0048-5

[B7] Barnes M. E. Evans E. M. Hazel A. Brownell S. E. Nesse R. M. (2017). Teleological reasoning, not acceptance of evolution, impacts students’ ability to learn natural selection. *Evol. : Educ. Outreach* 10:7. 10.1186/s12052-017-0070-6

[B8] Bartov H. (1981). Teaching students to understand the advantages and disadvantages of teleological and anthropomorphic statements in biology. *J. Res. Sci. Teach.* 18 79–86. 10.1002/tea.3660180113

[B9] Beck D. M. (2010). The appeal of the brain in the popular press. *Perspect. Psychol. Sci.* 5 762–766. 10.1177/1745691610388779 26161889

[B10] Bohner G. Dickel N. (2011). Attitudes and attitude change. *Annu. Rev. Psychol.* 62 391–417. 20809791 10.1146/annurev.psych.121208.131609

[B11] Boucher L. (2014). *Individual Differences Influence the Degree of Source Expertise Bias in Syllogistic Reasoning.* Master’s dissertation. Ottawa: Carleton University.

[B12] Bradac J. J. Desmond R. J. Murdock J. I. (1977). Diversity and density: lexically determined evaluative and informational consequences of linguistic complexity. *Commun. Monogr.* 44 273–283. 10.1080/03637757709390139

[B13] Brem S. K. Rips L. J. (2000). Explanation and evidence in informal argument. *Cogn. Sci.* 24 573–604. 10.1207/s15516709cog2404_2

[B14] Bromme R. Kienhues D. Porsch T. (2011). “Who knows what and who can we believe? Epistemological beliefs are beliefs about knowledge (mostly) to be attained from others,” in *Personal Epistemology in the Classroom: Theory, Research, and Implications for Practice*, eds Bendixen L. D. Haerle F. C. (Cambridge: Cambridge University Press), 163–193. 10.1017/cbo9780511691904.006

[B15] Burns A. I. Biswas A. Babin L. L. (1993). The operation of mental imagery as a mediator of advertising effects. *J. Adv.* 22 71–85. 10.1080/00913367.1993.10673405

[B16] Caramazza A. McCloskey M. Green B. (1981). Naive beliefs in “sophisticated” subjects: misconceptions about trajectories of objects. *Cognition* 9 117–123. 10.1016/0010-0277(81)90007-x 7196821

[B17] Carey S. (1985). Are children fundamentally different kinds of thinkers and learners than adults? *Think. Learn. Skills* 2 485–517.

[B18] Carpenter C. J. (2010). A meta-analysis of the effectiveness of health belief model variables in predicting behavior. *Health Commun.* 25 661–669. 10.1080/10410236.2010.521906 21153982

[B19] Chaiken S. (1980). Heuristic versus systematic information processing and the use of source versus message cues in persuasion. *J. Personal. Soc. Psychol.* 39 752–766. 10.1037/0022-3514.39.5.752

[B20] Chaiken S. Ledgerwood A. (2012). “A theory of heuristic and systematic information processing,” in *Handbook of Theories of Social Psychology*, eds van Lange P. A. M. Kruglanski A. W. Higgins E. T. (Thousand Oaks, CA: Sage), 246–266. 10.4135/9781446249215.n13

[B21] Chaiken S. Trope Y. (1999). *Dual-Process Models in Social Psychology.* New York, NY: Guilford.

[B22] Chen S. Chaiken S. (1999). “The heuristic-systematic model in its broader context,” in *Dual-Process Theories in Social Psychology*, eds Chaiken S. Trope Y. (New York, NY: Guilford Press), 73–96.

[B23] Chi M. T. H. Feltovich P. Glaser R. (1981). Categorization and representation of physics problems by experts and novices. *Cogn. Sci.* 5 121–152. 10.1207/s15516709cog0502_2

[B24] Cialdini R. Goldstein N. J. (2004). Social influence: conformity and compliance. *Annu. Rev. Psychol.* 55 591–621. 10.1146/annurev.psych.55.090902.142015 14744228

[B25] Cialdini R. B. (1984/2007). *Influence: Science and Practice*, 3rd Edn. New York, NY: HarperCollins.

[B26] Colombo M. (2017). Experimental philosophy of explanation rising: the case for a plurality of concepts of explanation. *Cogn. Sci.* 41 503–517. 10.1111/cogs.12340 26849022

[B27] Copeland D. E. Gunawan K. Bies-Hernandez J. B. (2011). Source credibility and syllogistic reasoning. *Mem. Cogn.* 39 117–127. 10.3758/s13421-010-0029-0 21264611

[B28] Cowan N. (2001). The magical number 4 in short-term memory: a reconsideration of mental storage capacity. *Behav. Brain Sci.* 24 87–114. 10.1017/s0140525x01003922 11515286

[B29] Craik K. J. W. (1952). *The Nature of Explanation.* Cambridge: CUP Archive.

[B30] Crano W. D. Prislin R. (2006). Attitudes and persuasion. *Annu. Rev. Psychol.* 57 345–374. 16318599 10.1146/annurev.psych.57.102904.190034

[B31] Craver C. F. (2014). “The ontic conception of scientific explanation,” in *Explanation in the Special Sciences: Explanation in the Biological and Historical Sciences*, eds Hütteman A. Kaiser M. (Dordrecht: Springer), 27–52. 10.1007/978-94-007-7563-3_2

[B32] Dacey M. (2017). Anthropomorphism as cognitive bias. *Philos. Sci.* 84 1152–1164. 10.1086/694039

[B33] Dennett D. (1987). *The Intentional Stance.* Cambridge: MIT Press.

[B34] Douven I. Schupbach J. N. (2015). The role of explanatory considerations in updating. *Cognition* 142 299–311. 10.1016/j.cognition.2015.04.017 26069937

[B35] Drummond C. Fischhoff B. (2017). Individuals with greater science literacy and education have more polarized beliefs on controversial science topics. *Proc. Natl. Acad. Sci. U.S.A.* 114 9587–9592. 10.1073/pnas.1704882114 28827344 PMC5594657

[B36] Dunbar K. (2001). “The analogical paradox: why analogy is so easy in naturalistic settings yet so difficult in the psychological laboratory,” in *The Analogical Mind: Perspectives from Cognitive Science*, eds Gentner D. Holyoak K. J. Kokinov B. N. (Cambridge, MA: The MIT Press), 313–334.

[B37] Eagly A. H. Chaiken S. (2005). “Attitude research in the 21st Century: the current state of knowledge,” in *The Handbook of Attitudes*, eds Albarracín D. Johnson B. T. Zanna M. P. (New Jersey: Lawrence Erlbaum Associates Publishers), 743–767.

[B38] Emmott R. (2020). *Russia Deploying Coronavirus Disinformation To Sow Panic in West, Eu Document Says.* London: Reuters.

[B39] Evans J. S. B. T. Barston J. Pollard P. (1983). On the conflict between logic and belief in syllogistic reasoning. *Mem. Cogn.* 11 295–306. 10.3758/bf03196976 6621345

[B40] Evans J. S. B. T. Curtis-Holmes J. (2005). Rapid responding increases belief bias: evidence for the dual-process theory of reasoning. *Think. Reason.* 11 382–389. 10.1080/13546780542000005

[B41] Frass M. Strassl R. P. Friehs H. Müllner M. Kundi M. Kaye A. D. (2012). Use and acceptance of complementary and alternative medicine among the general population and medical personnel: a systematic review. *Ochsner J.* 12 45–56. 22438782 PMC3307506

[B42] French L. Garry M. Mori K. (2011). Relative – not absolute – judgments of credibility affect susceptibility to misinformation conveyed during discussion. *Acta Psychol.* 136 119–128. 10.1016/j.actpsy.2010.10.009 21112042

[B43] Gelman S. A. Legare C. H. (2011). Concepts and folk theories. *Annu. Rev. Anthropol.* 40 379–398. 10.1146/annurev-anthro-081309-145822 23436950 PMC3579644

[B44] Gentner D. Holyoak K. J. Kokinov B. N. (2001). *The Analogical Mind: Perspectives From Cognitive Science.* Cambridge: MIT Press.

[B45] Gick M. L. Holyoak K. J. (1983). Schema induction and analogical transfer. *Cogn. Psychol.* 15 1–38. 10.1016/0010-0285(83)90002-6

[B46] Gilovich T. Griffin D. Kahneman D. (eds) (2002). *Heuristics and Biases: The Psychology of Intuitive Judgment.* Cambridge: Cambridge University Press.

[B47] Glasman L. R. Albarracín D. (2006). Forming attitudes that predict future behavior: a meta-analysis of the attitude-behavior relation. *Psychol. Bull.* 132 778–822. 10.1037/0033-2909.132.5.778 16910754 PMC4815429

[B48] Glennan S. S. (2009). Productivity, relevance and natural selection. *Biol. Philos.* 24 325–339. 10.1007/s10539-008-9137-7

[B49] Goethals G. R. Darley J. M. (1977). “Social comparison theory: an attributional approach,” in *Social Comparison Processes: Theoretical and Empirical Perspectives*, eds Suls J. M. Miller R. L. (Washington, DC: Hemisphere/Halsted).

[B50] Goldberg R. F. Thompson-Schill S. L. (2009). Developmental “roots” in mature biological knowledge. *Psychol. Sci.* 20 480–487. 10.1111/j.1467-9280.2009.02320.x 19399979 PMC3025485

[B51] Haig B. D. (2005). An abductive theory of scientific method. *Psychol. Methods* 10 371–388. 10.1037/1082-989x.10.4.371 16392993

[B52] Halford G. S. Wilson W. H. Phillips S. (2010). Relational knowledge: the foundation of higher cognition. *Trends Cogn. Sci.* 14 497–505. 10.1016/j.tics.2010.08.005 20884275

[B53] Hempel C. Oppenheim P. (1948). Studies in the logic of explanation. *Philos. Sci.* 15 135–175. 10.1086/286983

[B54] Hirschfield L. A. Gelman S. A. (1994). *Mapping the Mind: Domain Specificity in Cognition and Culture.* New York, NY: Cambridge University Press.

[B55] Hitt R. Perrault E. Smith S. Keating D. M. Nazione S. Silk K. (2016). Scientific message translation and the heuristic systematic model: insights for designing educational messages about progesterone and breast cancer risks. *J. Cancer Educ.* 31 389–396. 10.1007/s13187-015-0835-y 25903053 PMC5501319

[B56] Hobeika L. Diard-Detoeuf C. Garcin B. Levy R. Volle E. (2016). General and specialized brain correlates for analogical reasoning: a meta-analysis of functional imaging studies. *Hum. Brain Mapp.* 37 1953–1969. 10.1002/hbm.23149 27012301 PMC6867453

[B57] Hook C. J. Farah M. J. (2013). Look again: effects of brain images and mind–brain dualism on lay evaluations of research. *J. Cogn. Neurosci.* 25 1397–1405. 10.1162/jocn_a_00407 23879877

[B58] Hubbard T. L. Ruppel S. E. (2013). Ratings of causality and force in launching and shattering. *Vis. Cogn.* 21 987–1009. 10.1080/13506285.2013.847883

[B59] Johnson B. T. Eagly A. H. (1989). The effect of involvement on persuasion: a meta-analysis. *Psychol. Bull.* 106 290–314. 10.1037/0033-2909.106.2.290

[B60] Johnson S. C. (2000). The recognition of mentalistic agents in infancy. *Trends Cogn. Sci.* 4 22–28. 10.1016/s1364-6613(99)01414-x 10637619

[B61] Johnson-Laird P. N. (1983). *Mental Models: Towards a Cognitive Science of Language, Inference, and Consciousness.* Cambridge, MA: Harvard University Press.

[B62] Johnson-Laird P. N. Byrne R. M. J. (2002). Conditionals: a theory of meaning, pragmatics, and inference. *Psychol. Rev.* 109 646–678. 10.1037/0033-295x.109.4.646 12374323

[B63] Johnson-Laird P. N. Steedman M. (1978). The psychology of syllogisms. *Cogn. Psychol.* 10 64–99. 10.1016/0010-0285(78)90019-1

[B64] Kahneman D. Tversky A. (1972). Subjective probability: a judgment of representativeness. *Cogn. Psychol.* 3 430–454. 10.1016/0010-0285(72)90016-3

[B65] Kaptchuk T. J. Eisenberg D. M. (1998). The persuasive appeal of alternative medicine. *Ann. Int. Med.* 129 1061–1065. 10.7326/0003-4819-129-12-199812150-00011 9867762

[B66] Keil F. C. (2006). Explanation and understanding. *Annu. Rev. Psychol.* 57 227–254. 16318595 10.1146/annurev.psych.57.102904.190100 PMC3034737

[B67] Keil F. C. Wilson R. A. (2001). *Explanation and Cognition.* Cambridge: MIT Press.

[B68] Kelemen D. (1999). Function, goals and intention: children’s teleological reasoning about objects. *Trends Cogn. Sci.* 3 461–468. 10.1016/s1364-6613(99)01402-3 10562725

[B69] Keren G. (1991). Calibration and probability judgments: conceptual and methodological issues. *Acta Psychol.* 77 217–273. 10.1016/0001-6918(91)90036-y

[B70] Khemlani S. Johnson-Laird P. N. (2012). Theories of the syllogism: a meta-analysis. *Psychol. Bull.* 138 427–457. 10.1037/a0026841 22289108

[B71] Klauer K. C. Musch J. Naumer B. (2000). On belief bias in syllogistic reasoning. *Psychol. Rev.* 107 852–884. 10.1037/0033-295x.107.4.852 11089409

[B72] Koehler D. J. (1991). Explanation, Imagination, and Confidence in Judgment. *Psychol. Bull.* 110 499–519. 10.1037/0033-2909.110.3.499 1758920

[B73] Kolstø S. D. (2001). Scientific literacy for citizenship: tools for dealing with the science dimension of controversial socioscientific issues. *Sci. Educ.* 85 291–310. 10.1002/sce.1011

[B74] Koriat A. Ma’ayan H. (2005). The effects of encoding fluency and retrieval fluency on judgments of learning. *J. Mem. Lang.* 52 478–492. 10.1016/j.jml.2005.01.001

[B75] Kraft P. W. Lodge M. Taber C. S. (2015). Why people “don’t trust the evidence”: motivated reasoning and scientific beliefs. *Ann. Am. Acad. Political Soc. Sci.* 658 121–133. 10.1177/0002716214554758

[B76] Kruger J. Dunning D. (1999). Unskilled and unaware of it: how difficulties in recognizing one’s own incompetence lead to inflated self-assessments. *J. Personal. Soc. Psychol.* 77 1121–1134. 10.1037/0022-3514.77.6.1121 10626367

[B77] Krull D. S. Silvera D. H. (2013). The stereotyping of science: superficial details influence perceptions of what is scientific. *J. Appl. Soc. Psychol.* 43, 1660–1667. 10.1111/jasp.12118

[B78] Kvidera S. Koutstaal W. (2008). Confidence and decision type under matched stimulus conditions: overconfidence in perceptual but not conceptual decisions. *J. Behav. Decis. Mak.* 21 253–281. 10.1002/bdm.587

[B79] Kyllonen P. C. Christal R. E. (1990). Reasoning ability is little more working-memory capacity?! *Intelligence* 14 389–433. 10.1016/s0160-2896(05)80012-1

[B80] Lichtenstein S. Fischhoff B. (1977). Do those who know more also know more about how much they know? *Organ. Behav. Hum. Perform.* 20 159–183. 10.1016/0030-5073(77)90001-0

[B81] Lombrozo T. (2007). Simplicity and probability in causal explanation. *Cogn. Psychol.* 55 232–257. 10.1016/j.cogpsych.2006.09.006 17097080

[B82] Lombrozo T. (2012). “Explanation and abductive inference,” in *Oxford Handbook of Thinking and Reasoning*, eds Holyoak K. J. Morrison R. G. (Oxford: Oxford University Press), 260–276.

[B83] Lombrozo T. Vasilyeva N. (2017). “Causal explanation,” in *Oxford Handbook of Causal Reasoning*, ed. Waldmann M. (Oxford: Oxford University Press), 415–432.

[B84] Mandler J. M. McDonough L. (1993). Concept formation in infancy. *Cogn. Dev.* 8 291–318. 10.1016/s0885-2014(93)80003-c

[B85] Mandler J. M. McDonough L. (1998). On developing a knowledge base in infancy. *Dev. Psychol.* 34 1274–1288. 10.1037/0012-1649.34.6.1274 9823512

[B86] Markovits H. Nantel G. (1989). The belief-bias effect in the production and evaluation of logical conclusions. *Mem. Cogn.* 17 11–17. 10.3758/bf03199552 2913452

[B87] McAfee E. A. Proffitt D. R. (1991). Understanding the surface orientation of liquids. *Cogn. Psychol.* 23 483–514. 10.1016/0010-0285(91)90017-i

[B88] McCabe D. P. Castel A. D. (2008). Seeing is believing: The effect of brain images on judgments of scientific reasoning. *Cognition* 107 343–352. 10.1016/j.cognition.2007.07.017 17803985

[B89] McCloskey M. (1983). “Naïve theories of motion,” in *Mental Models*, eds Gentner D. Stevens A. (New Jersey: Erlbaum), 299–324.

[B90] McCloskey M. Kohl D. (1983). Naive physics: the curvilinear impetus principle and its role in interactions with moving objects. *J. Exp. Psychol.* 9 146–156. 10.1037/0278-7393.9.1.146 6220112

[B91] McGinnes E. Ward C. (1980). Better liked than right: trustworthiness and expertise in credibility. *Personal. Soc. Psychol. Bull.* 6 467–472. 10.1177/014616728063023

[B92] Medin D. L. Atran S. (eds) (1999). *Folkbiology.* Cambridge: MIT Press.

[B93] Michael R. B. Newman E. J. Vuorre M. Cumming G. Garry M. (2013). On the (non)persuasive power of a brain image. *Psychon. Bull. Rev.* 20 720–725. 10.3758/s13423-013-0391-6 23400855

[B94] Miller J. (1984). Culture and the development of everyday social explanation. *J. Personal. Soc. Psychol.* 46 961–978. 10.1037/0022-3514.46.5.961 6737211

[B95] Miller P. H. Aloise P. A. (1989). Young children’s understanding of the psychological causes of behaviour. *Child Dev.* 60 257–285. 10.2307/1130975 28661004

[B96] Mills J. Jellison J. M. (1967). Effect on opinion change of how desirable the communication is to the audience the communicator addressed. *J. Personal. Soc. Psychol.* 6 98–101. 10.1037/h0021217 6032764

[B97] Motta M. Callaghan T. Sylvester S. (2018). Knowing less but presuming more: dunning-Kruger effects and the endorsement of anti-vaccine policy attitudes. *Soc. Sci. Med.* 211 274–281. 10.1016/j.socscimed.2018.06.032 29966822

[B98] Murphy G. L. (2000). “Explanatory concepts,” in *Explanation and Cognition*, eds Keil F. C. Wilson R. A. (Cambridge: MIT Press.), 361–392.

[B99] Nersessian N. (1999). “Model-based reasoning in conceptual change,” in *Model-Based Reasoning in Scientific Discovery*, eds Magani L. Nersessian N. Thagard P. (New York, NY: Kluwer/Plenum), 5–22. 10.1007/978-1-4615-4813-3_1

[B100] Newhagen J. Nass C. (1988). Defining and measuring credibility for evaluating credibility of newspapers and TV news. *Journal. Q.* 66 277–284. 10.1177/107769908906600202

[B101] Newman I. R. Gibb M. Thompson V. A. (2017). Rule-based reasoning is fast and belief-based reasoning can be slow: challenging current explanations of belief-bias and base-rate neglect. *J. Exp. Psychol.* 43 1154–1170. 10.1037/xlm0000372 28191989

[B102] Newstead S. E. Evans J. B. T. (1993). Mental models as an explanation of belief bias effects in syllogistic reasoning. *Cognition* 46 93–97. 10.1016/0010-0277(93)90024-p 1490324

[B103] Nisbett R. E. (2003). *The Geography of Thought: How Asians and Westerners Think Differently…and Why.* New York, NY: Free Press.

[B104] Oakhill J. V. Johnson-Laird P. N. Garnham A. (1989). Believability and syllogistic reasoning. *Cognition* 31 117–140. 10.1016/0010-0277(89)90020-6 2721132

[B105] O’Connor C. Rees G. Joffe H. (2012). Neuroscience in the public sphere. *Neuron* 74 220–226. 22542177 10.1016/j.neuron.2012.04.004

[B106] Pacer M. Lombrozo T. (2017). Ockham’s razor cuts to the root: simplicity in causal explanation. *J. Exp. Psychol.* 146 1761–1780. 10.1037/xge0000318 29251989

[B107] Park S. Y. Constantino N. Yun G. W. Moser L. Cortes-Arriola D. (2020). US College Students’ marijuana information sources, confidence in knowledge, and objective knowledge. *J. Health Commun.* 25 859–869. 10.1080/10810730.2020.1840677 33151134

[B108] Paulhus D. L. Harms P. D. Bruce M. N. Lysy D. C. (2003). The over-claiming technique: measuring self-enhancement independent of ability. *J. Personal. Soc. Psychol.* 84 890–904. 10.1037/0022-3514.84.4.890 12703655

[B109] Petty R. E. Fleming M. A. White P. H. (1999). Stigmatized sources and persuasion: prejudice as a determinant of argument scrutiny. *J. Personal. Soc. Psychol.* 76 19–34. 10.1037/0022-3514.76.1.19 9972550

[B110] Petty R. E. Wegner D. T. Fabrigar L. W. (1997). Attitudes and attitude change. *Annu. Rev. Psychol.* 48 609–647. 9046570 10.1146/annurev.psych.48.1.609

[B111] Petty R. E. Cacioppo J. T. (1986). “The elaboration likelihood model of persuasion,” in *Advances in Experimental Social Psychology*, ed. Berkowitz L. (New York, NY: Academic Press), 123–205.

[B112] Pew Research Center (2015). *Public and Scientists’ Views on Science and Society.* Avaliable at: http://www.pewinternet.org/files/2015/01/PI_ScienceandSociety_Report_012915.pdf (accessed March 01, 2015).

[B113] Poling D. A. Evans E. M. (2002). Why do birds of a feather flock together? Developmental change in the use of multiple explanations: intention, teleology and essentialism. *Br. J. Dev. Psychol.* 20 89–112. 10.1348/026151002166343

[B114] Pornpitakpan C. (2004). The persuasiveness of source credibility: a critical review of five decades’ evidence. *J. Appl. Soc. Psychol.* 34 243–281. 10.1111/j.1559-1816.2004.tb02547.x

[B115] Poulin-Dubois D. (1999). “Infants’ distinction between animate and inanimate objects: the origins of naïve psychology,” in *Early Social Cognition: Understanding Others in the First Months of Life*, ed. Rochat P. (Hillsdale: Lawrence Erlbaum Associates), 257–280.

[B116] Proffitt D. R. Gilden D. L. (1989). Understanding natural dynamics. *J. Exp. Psychol.* 15 384–393. 2525605

[B117] Rehder B. Burnett R. C. (2005). Feature inference and the causal structure of categories. *Cogn. Psychol.* 50 274–314. 10.1016/j.cogpsych.2004.09.002 15826612

[B118] Rehder B. Kim S. W. (2010). Causal status and coherence in causal-based categorization. *J. Exp. Psychol.* 36 1171–1206. 10.1037/a0019765 20804292

[B119] Rhine R. J. Severance L. J. (1970). Ego-involvement, discrepancy, source credibility, and attitude change. *J. Personal. Soc. Psychol.* 16 175–190. 10.1037/h0029832

[B120] Rhodes R. E. Rodriguez F. Shah P. (2014). Explaining the alluring influence of neuroscience information on scientific reasoning. *J. Exp. Psychol.* 40 1432–1440. 10.1037/a0036844 24820673

[B121] Richland L. E. Chan T. K. Morrison R. G. Au T. K. F. (2010). Young children’s analogical reasoning across cultures: similarities and differences. *J. Exp. Child Psychol.* 105 146–153. 10.1016/j.jecp.2009.08.003 19896676

[B122] Robins-Early N. (2020). *Coronavirus Misinformation Is Spreading Through Bogus Texts and Group Chats.* New York, NY: The Huffing Post.

[B123] Rossiter J. Percy L. (1978). “Visual imaging ability as a mediator of advertising response,” in *Advances in Consumer Research*, Vol. 5 ed. Hunt H. K. (Ann Arbor: Association for Consumer Research), 621–629.

[B124] Rumelhart D. E. Norman D. A. (1981). “Analogical processes in learning,” in *Learning and Cognition*, ed. Anderson J. R. (Hillsdale, N.J: Lawrence Erlbaum Associates), 335–359.

[B125] Sa W. West R. F. Stanovich K. E. (1999). The domain specificity and generality of belief bias: searching for generalizable critical thinking skills. *J. Educ. Psychol.* 91 497–510. 10.1037/0022-0663.91.3.497

[B126] Salmon W. (1989). *Four Decades of Scientific Explanation.* Minneapolis, MN: University of Minnesota Press.

[B127] Scharrer L. Bromme R. Britt M. A. Stadtler M. (2012). The seduction of easiness: how science depictions influence laypeople’s reliance on their own evaluation of scientific information. *Learn. Instr.* 22 231–243. 10.1016/j.learninstruc.2011.11.004

[B128] Schlosser A. E. (2003). Experiencing products in a virtual world: the role of goals and imagery in influencing attitudes versus intentions. *J. Consum. Res.* 30 184–196. 10.1086/376807

[B129] Schoenherr J. Thomson R. Davies J. (2011). “What makes an explanation believable?: mechanistic and anthropomorphic explanations of natural phenomena,” in *Proceedings of the Annual Meeting of the Cognitive Science Society*, Vol. 33 Boston, MA.

[B130] Schoenherr J. R. Lacroix G. L. (2020). Performance monitoring during categorization with and without prior knowledge: a comparison of confidence calibration indices with the certainty criterion. *Can. J. Exp. Psychol.* 74 302–315. 10.1037/cep0000199 31971435

[B131] Schoenherr J. R. Leth-Steensen C. Petrusic W. M. (2010). Selective attention and subjective confidence calibration. *Attent. Percept. Psychophys.* 72 353–368. 10.3758/app.72.2.353 20139451

[B132] Schulte G. (2020). *Poll: 66 Percent of Americans Say Coronavirus Will Not Impact Personal Life, Despite Growing Anxiety Worldwide.* Washington, D.C: The Hill. Available online at: https://thehill.com/hilltv/rising/483017-poll-amid-growing-anxiety-worldwide-66-of-americans-say-coronavirus-will-not (accessed February 17, 2020).

[B133] Schwarz N. Vaughn L. A. (2002). “The availability heuristic revisited: ease of recall and content of recall as distinct sources of information,” in *Heuristics and Biases: The Psychology of Intuitive Judgment*, eds Gilovich T. Griffin D. Kahneman D. (Cambridge: Cambridge University Press), 103–119. 10.1017/cbo9780511808098.007

[B134] Shynkaruk J. M. Thompson V. A. (2006). Confidence and accuracy in deductive reasoning. *Mem. Cogn.* 34 619–632. 10.3758/bf03193584 16933770

[B135] Slater M. D. Rouner D. (1996). How message evaluation and source attributes may influence credibility assessment and belief change. *Journal. Mass Commun. Q.* 73 974–991. 10.1177/107769909607300415

[B136] Sloman S. A. (1994). When explanations compete: the role of explanatory coherence on judgments of likelihood. *Cognition* 52 1–21. 10.1016/0010-0277(94)90002-7 7924197

[B137] Sparks J. R. Rapp D. N. (2011). Readers’ reliance on source credibility in the service of comprehension. *J. Exp. Psychol.* 37 230–247. 10.1037/a0021331 21244116

[B138] Spelke E. S. Kinzler K. D. (2007). Core knowledge. *Dev. Sci.* 10 89–96. 17181705 10.1111/j.1467-7687.2007.00569.x

[B139] Stanovich K. E. (2003). “The fundamental computational biases of human cognition: heuristics that (sometimes) impair decision making and problem solving,” in *The Psychology of Problem Solving*, eds Davidson J. E. Sternberg R. J. (New York, NY: Cambridge University Press), 291–342. 10.1017/cbo9780511615771.011

[B140] Stanovich K. E. (2004). *The Robot’s Rebellion: Finding Meaning in the Age of Darwin.* Chicago, IL: University of Chicago Press.

[B141] Stanovich K. E. West R. F. (2000). Individual differences in reasoning: implications for the rationality debate? *Behav. Brain Sci.* 23 645–665. 10.1017/s0140525x00003435 11301544

[B142] Stanovich K. E. West R. F. (2007). Natural myside bias is independent of cognitive ability. *Think. Reason.* 13 225–247. 10.1080/13546780600780796

[B143] Stanovich K. E. West R. F. (2008). On the relative independence of thinking biases and cognitive ability. *J. Personal. Soc. Psychol.* 94 672–695. 10.1037/0022-3514.94.4.672 18361678

[B144] Strevens M. (2008). *Depth: An Account of Scientific Explanation.* Cambridge, MA: Harvard University Press.

[B145] Süβ H.-M. Oberauer K. Wittmann W. W. Wilhelm O. Schulze R. (2002). Working-memory capacity explains reasoning ability—and a little bit more. *Intelligence* 30 261–288. 10.1016/s0160-2896(01)00100-3

[B146] Talanquer V. (2007). Explanations and teleology in chemistry education. *Int. J. Sci. Educ.* 29 853–870. 10.1080/09500690601087632

[B147] Talanquer V. (2010). Exploring dominant types of explanations built by general chemistry students. *Int. J. Sci. Educ.* 32 2393–2412. 10.1080/09500690903369662

[B148] Tamir P. Zohar A. (1991). Anthropomorphism and teleology in reasoning about biological phenomena. *Sci. Educ.* 75 57–67. 10.1002/sce.3730750106

[B149] Thomm E. Bromme R. (2012). “It should at least seem scientific!” Textual features of “scientificness” and their impact on lay assessments of online information. *Sci. Educ.* 96 187–211. 10.1002/sce.20480

[B150] Thulin S. Pramling N. (2009). Anthropomorphically speaking: on communication between teachers and children in early childhood biology education. *Int. J. Early Years Educ.* 17 137–150. 10.1080/09669760902982331

[B151] Trippas D. Thompson V. A. Handley S. J. (2017). When fast logic meets slow belief: evidence for a parallel-processing model of belief bias. *Mem. Cogn.* 45 539–552. 10.3758/s13421-016-0680-1 28028779 PMC5432582

[B152] Trout J. D. (2007). The psychology of scientific explanation. *Philos. Compass* 3 564–591.

[B153] Trout J. D. (2008). Seduction without cause: uncovering explanatory neurophilia. *Trends Cogn. Sci.* 12 281–282. 10.1016/j.tics.2008.05.004 18606564

[B154] Tversky A. Kahneman D. (1973). Availability: a heuristic for judging frequency and probability. *Cogn. Psychol.* 5 207–232. 10.1016/0010-0285(73)90033-9

[B155] Vosniadou S. Ortony A. (eds) (1989). *Similarity and Analogical Reasoning.* Cambridge: Cambridge University Press.

[B156] Waxman S. Medin D. Ross N. (2007). Folkbiological reasoning from a cross-cultural developmental perspective: early essentialist notions are shaped by cultural beliefs. *Dev. Psychol.* 43 294–308. 10.1037/0012-1649.43.2.294 17352540

[B157] Weisberg D. S. Keil F. C. Goodstein J. Rawson E. Gray J. R. (2008). The seductive allure of neuroscience explanations. *J. Cogn. Neurosci.* 20 470–477. 10.1162/jocn.2008.20040 18004955 PMC2778755

[B158] Wellman H. M. (2011). “Developing a theory of mind,” in *Handbook of Childhood Cognitive Development*, 2nd Edn, ed. Goswami U. (Oxford: Blackwell).

[B159] Wellman H. M. Cross D. Watson J. (2001). Meta-analysis of theory-of-mind development: the truth about false belief. *Child Dev.* 72 655–684. 10.1111/1467-8624.00304 11405571

[B160] Wilson E. J. Sherrell D. L. (1993). Source effects in communication and persuasion research: a meta-analysis of effect size. *J. Acad. of Market. Sci.* 21 101–112. 10.1007/bf02894421

[B161] Wison T. D. Lindsey S. Schooler T. Y. (2000). A model of dual attitudes. *Psychol. Rev.* 107 101–126. 10.1037/0033-295x.107.1.101 10687404

[B162] Wolff P. (2007). Representing causation. *J. Exp. Psychol.* 136 82–111. 10.1037/0096-3445.136.1.82 17324086

[B163] Woodward J. (2003). *Making Things Happen: A Theory of Causal Explanation.* Oxford: Oxford University Press.

[B164] Zhu D. Xie X. Gan Y. (2011). Information source and valence: how information credibility influences earthquake risk perception. *J. Environ. Psychol.* 31 129–136. 10.1016/j.jenvp.2010.09.005

[B165] Zimmerman C. Bisanz G. L. Bisanz J. Klein J. S. Klein P. (2001). Science at the supermarket: a comparison of what appears in the popular press, experts’ advice to readers, and what students want to know. *Public Underst. Sci.* 10 37–58.

[B166] Ziori E. Dienes Z. (2008). How does prior knowledge affect implicit and explicit concept learning? *Q. J. Exp. Psychol.* 61 601–624. 10.1080/17470210701255374 18938278

